# Stillbirth of a mandrill (*Mandrillus sphinx*) in the wild: perinatal behaviors and delivery sequences

**DOI:** 10.1007/s10329-023-01112-6

**Published:** 2023-12-22

**Authors:** Berta Roura-Torres, Paul Amblard-Rambert, Pascal Lepou, Peter M. Kappeler, Marie J. E. Charpentier

**Affiliations:** 1 Behavioral Ecology and Sociobiology Unit, German Primate Center, Leibniz Institute for Primate Research, Kellnerweg 4, 37007 Göttingen, Germany; 2 Department of Sociobiology/Anthropology, Institute of Zoology and Anthropology, Johann-Friedrich-Blumenbach, Georg-August University Göttingen, Kellnerweg 6, 37077 Göttingen, Germany; 3 Projet Mandrillus, Parc de la Lékédi, BP 52, Bakoumba, Gabon; 4 ISEM, UMR5554–University of Montpellier/CNRS/IRD/EPHE, Place Eugène Bataillon (cc065), 34905 Montpellier, France; 5 Department for the Ecology of Animal Societies, Max Planck Institute of Animal Behavior, Bücklestraβe 5, 78467 Constance, Germany

**Keywords:** Reproductive failure, Stillbirth, Parturition behavior, Reproduction, *Mandrillus sphinx*

## Abstract

Birth is a fundamental event in the life of animals, including our own species. More reports of wild non-human primate births and stillbirths are thus needed to better understand the evolutionary pressures shaping parturition behaviors in our lineage. In diurnal non-human primates, births generally occur at night, when individuals are resting. Consequently, they are difficult to observe in the wild and most of the current knowledge regarding perinatal behaviors comes from rare daytime births. Information about stillbirths is even rarer and their proximate causes are generally unknown. Here, we present detailed observations of a daytime birth of a stillborn wild mandrill (*Mandrillus sphinx*). During this event, which lasted an entire day, we recorded the behaviors of the parturient female ad libitum, using video recordings and photos. The 5-year-old female was primiparous and of low dominance rank. The length of her pregnancy was shorter than usual and the partum phase was extremely long compared to other birth reports in non-human primates. The female disappeared shortly after this event and was assumed to have died. We discuss the possible causes of this stillbirth including the infant’s presentation at birth and maternal inexperience.

**Supplementary Information:**

The online version contains supplementary material available at 10.1007/s10329-023-01112-6.

## Introduction

Birth is a key milestone in animals’ lives during which both the mother and the infant are extremely vulnerable. In non-human primates, these events are generally short and discrete and most of them take place at night (Jolly 1972), outside the visual range of researchers (e.g., nests in great apes or high in the trees or in tree holes in other species; Ding et al. 2013; Hirata et al. 2011; Yao et al. 2012). So far, 103 diurnal birthing events have been reported in 29 species of wild non-human primates (Table S1, Supplementary Material), including 9 stillbirths.

Stillbirth, defined as the birth of a fetus that died in the womb during late pregnancy (Saiyed et al. 2018; Sesbuppha et al. 2008), is a reproductive failure resulting from a deficiency of the materno–feto–placental unit to maintain an appropriate fetal environment (Beehner et al. 2006). In humans, stillbirths concerned 1.39% of worldwide births in 2021 (UN IGME 2023), and although this phenomenon is also presumably common in non-human primates living in captivity (e.g., 9.9% of conceptions in *Macaca* sp.: Small 1982; 4.82% of conceptions in *Papio hymadrayas*: Schlabritz-Loutsevitch et al. 2008; 10–13% of conceptions in great apes: Saiyed et al. 2018), it has been reported in only three different natural and non-provisioned populations (Table S1, Supplementary Materials). Moreover, the processes and causes of stillbirths are still poorly understood and most of the available data either come from humans or from captive non-human primates (Saiyed et al. 2018; Sesbuppha et al. 2008; Schlabritz-Loutsevitch et al. 2008; Small 1982). Given the critical relevance of births on animals’ reproductive outcomes, reports on stillbirths are essential to gain insights into the ecology of pregnancy and pregnancy failures. Moreover, they provide a unique framework for studying the maternal response to their infant’s death (Fernández-Fueyo et al. 2021). In January 2021, we witnessed a daytime parturition of a female mandrill (*Mandrillus sphinx*) that resulted in a stillbirth in a natural population. The parturient female disappeared two days after the delivery and was assumed to have died due to this pregnancy failure. We provide a detailed report of this event, including unique data on the associated perinatal behaviors in this wild non-human primate.

## Methods

The study population was founded by 65 captive-bred mandrills previously housed at the CIRMF (Centre International de Recherches Médicales de Franceville) and released in two waves in 2002 and 2006 (Peignot et al. 2008) in a natural area, in southern Gabon. Released females immediately started reproducing with wild migrant males and, in January 2021, when the stillbirth occurred, the group consisted of ca. 250 wild-born individuals with only seven captive-born females remaining. Excluding occasional trappings (Poirotte et al. 2017), this population has not been manipulated since 2012, when a long-term field project (the “Mandrillus Project”) began to study the socio-ecology of this little-known primate. The study group roams freely in the Lékédi Park (Bakoumba, Gabon) and its surroundings, and has been monitored daily. Field assistants collect detailed data on individual life history, social behaviors, group composition, and GPS locations. The exact date of birth is known for the majority of the individuals born since 2012, and for the remaining group members, age is estimated based on general condition and patterns of tooth eruption and wear (Galbany et al. 2014).

Female mandrills are philopatric and their reproductive state is recorded in this population on a daily basis (Dezeure et al. 2022). Mandrills are seasonal breeders with a conception and birth peak in July and January, respectively (Dezeure et al. 2022). Average gestation length is 175 days (range, 163–190 days, SD = 4.7,* N* = 103; Dezeure et al. 2022), and female average age at first birth is 4.82 years (range, 3.16–6.73 years, SD = 0.64,* N* = 52; unpublished data). Females develop a distinct sexual swelling during estrus that gradually inflates until reaching a maximal size around ovulation. A distinct pregnancy swelling also appears about two months following fertilization.

The parturient female was born in the group in 2016 and was thus about 5-year-old at the time of delivery. She was low-ranking and primiparous. Due to COVID-19 pandemic, we did not monitor her sexual swelling but pregnancy probably started around late July 2020 because of a clear pregnancy swelling observed in October 2020, when observers were back in the field. During the last month of pregnancy, she exhibited a black crust around her vulva (probably dried blood) during nine subsequent days, but her pregnancy swelling was unchanged and of normal shape and size. We did not detect any abnormal behaviors of the female during the days prior to delivery.

**Fig. 1:**
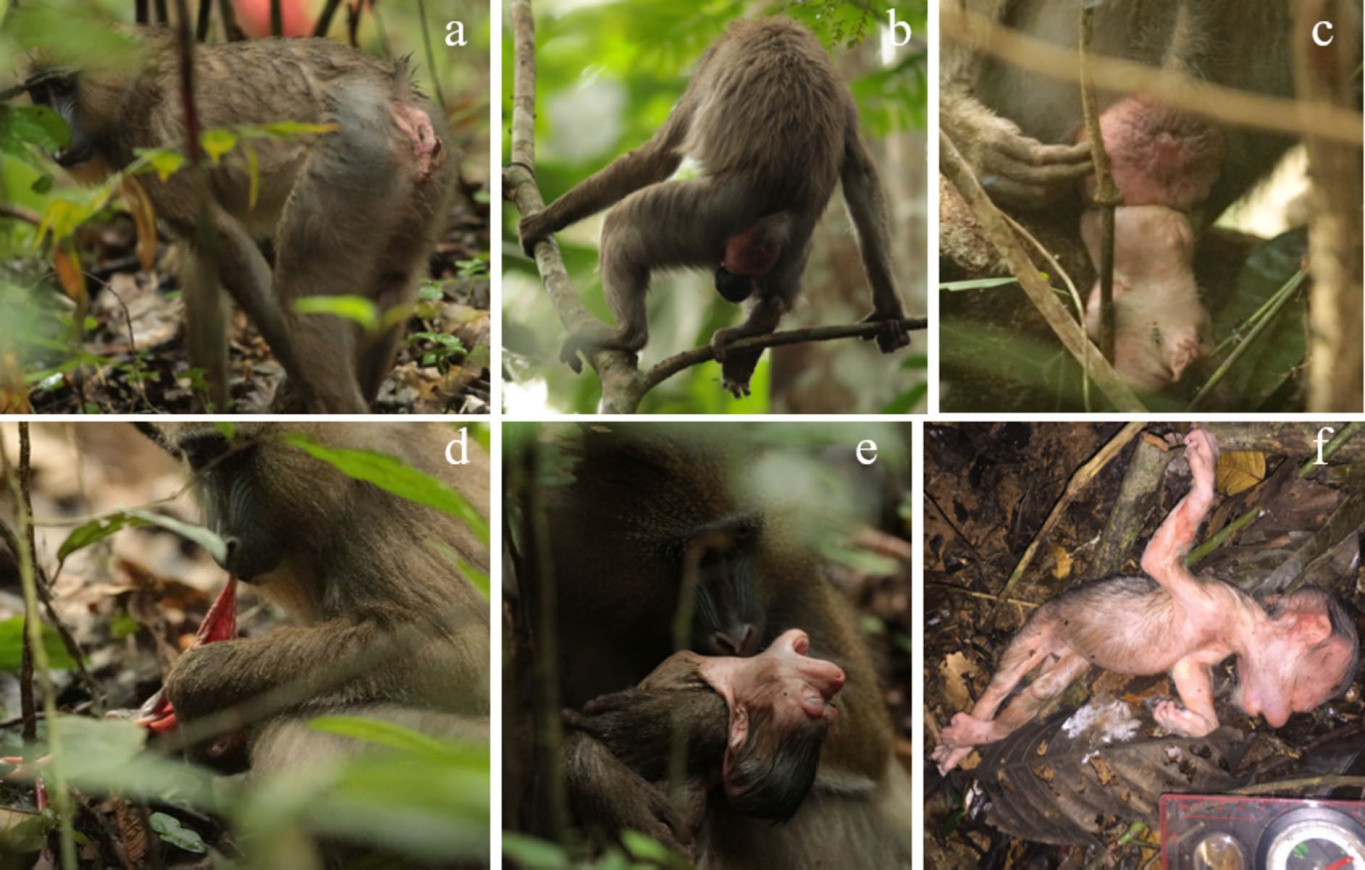
Photos of key events observed during a daytime parturition that resulted in a stillbirth: **a** Parturient female mandrill the morning of the day of parturition. **b** Parturient female undergoing a contraction. The crown of the infant’s head is visible. **c** Ongoing delivery with part of the infant’s body out. **d** Placenta’s consumption. **e** Parturient female holding and cleaning her infant’s corpse. **f** Corpse of the dead female infant

The sequence of events reported here took place on January 21, 2021 between 08:50 a.m. and 05:14 p.m., during our daily routine observations. The study group is extremely well habituated to human presence, we were therefore able to follow the parturient female closely without disturbing her, just before, during and after the delivery of her dead infant. We recorded her behaviors ad libitum through manual notes, photo and video recordings. For the description of the parturition and the timing of events, we used only data from video recordings and split events into three phases (Timmermans and Vossen 1996): prepartum or labor phase (which starts with the first contraction and ends when the head of the infant is visible), partum or birth phase (which starts when the head of the infant is visible and ends when the body has fully emerged) and postpartum phase (which starts after the birth of the infant and includes the complete consumption of the afterbirth). We then summarized these behaviors into ten categories (Table S2, Supplementary Material). We further calculated the infant’s crown-rump length (CRL) using a picture of the corpse next to a scale (Fig. 1f).

## Results

### Prepartum phase

The field team found the studied group at 07:29 a.m. on January 21, 2021. At 08:50 a.m., we located the parturient female who showed evidence of impeding birth as she was behaving unusually and was walking with a hunched back. She further showed a dilated vulva with a colorless fluid, presumably the amniotic fluid, dripping (Fig. 1a). Due to the steepness of the field and density of foliage, we lost the female at 09:02 a.m. and saw her again at 03:31 p.m. At that time, she was sitting on a branch at 1 m from the ground and was self-exploring her anogenital area. The fluid dripping from her vulva was bloody and flies surrounded her anogenital area. At 03:33 p.m., we witnessed, for the first time, a contraction. Contraction bouts were intermittent and on average, they lasted 3.42 s (range: 2–8 s, SD = 1.78, *N* = 12 contractions for a period of 41 s). The mean inter-contraction interval was 17.3 s (range: 1–60 s, SD = 21.83, *N* = 10 contractions for a period of 173 s). Contractions were accompanied by high-pitched grunts emitted by the parturient female.

During the prepartum phase, the female was often self-exploring her anogenital area or resting (Table 1). During self-exploring bouts, she grabbed and pulled her tail several times. When the female moved, she climbed to higher branches of the trees around her. The parturient female showed clear signs of tiredness and discomfort and tried to get rid of the flies around her. Although she was in the middle of the group, she did not exhibit any behavior that could suggest that she was seeking assistance from groupmates who also did not pay attention to her. This entire phase lasted 6 h and 56 min including 7 min and 32 s of video recording (Table 1).

**Table 1:** Duration of behaviors by birth phase

Behavior	Prepartum	Partum	Postpartum
	Duration	Duration	Duration
Carrying the infant	0	0	00:00:04
Cleaning the infant	0	0	00:02:54
Contraction	00:00:41	00:01:13	0
Exploration of umbilical cord	0	0	00:00:08
Foraging	0	00:00:04	0
Moving	00:00:08	00:01:47	00:00:14
Placentophagia	0	0	00:01:00
Resting	00:01:49	00:07:55	00:00:06
Self-exploring	00:04:54	00:07:55	00:01:19
Vigilance	0	0	00:01:33
Total duration	> 06:56:00	01:05:00	> 00:38:00
Video recording duration	00:07:32	00:18:54	00:07:18

### Partum phase

At 03:46 p.m. the infant’s crown became visible (Fig. 1b). At that stage, the parturient female moved to a higher branch and showed intermittent contraction bouts (average duration: 4.24 s, range: 1–14 s, SD = 3.53, *N* = 17 contractions for a period of 73 s). The mean inter-contraction interval was 6.5 s (range: 2–15 s, SD = 5.12, *N* = 4 contractions for a period of 26 s). During that stage, the female frequently self-explored her anogenital area. At 04:19 p.m. she moved back to the ground and foraged for 4 s, although she did not ingest anything. At 04:28 p.m. the infant’s head emerged from the birth canal in the vertex occiput anterior presentation (i.e., facing the backside of the mother), and at 04:31 p.m., shoulders were also observed (Fig. 1c). The infant was clearly dead, its eyes were closed and swollen and its mouth was opened. The parturient female then attempted unsuccessfully to pull the infant’s body out of the birth canal by pulling it through the neck. At 04:49 p.m., the infant’s upper body emerged and the umbilical cord became visible around the infant’s body. For 1 min and 46 s, the parturient female was out of sight, and when we saw her again at 04:51 p.m., she was sitting on the ground with the infant’s corpse on her side. The corpse was still attached to the umbilical cord, and she had not delivered the placenta yet.

The parturient female appeared tired and spent more time resting than during the previous phase (Table 1). Moreover, the rest of the group continued moving forward, she thus had to follow it. This entire phase lasted 1 h and 5 min including 18 min and 4 s of video recording (Table 1).

### Postpartum phase

Following birth, the female investigated her own anogenital area, and licked the umbilical cord and the infant’s corpse, especially around the mouth (Fig. 1e). She then moved by holding the corpse with her left hand for 4 s, left the corpse on the ground and continued moving without supporting it. The corpse was thus pulled and dragged by the umbilical cord during motion. At 04:53 p.m., she cleaned again her infant’s corpse for 4 min before moving again. At 05:01 p.m., the corpse was stuck against a root and the tension on the umbilical cord hastened the emergence of the placenta. She immediately started feeding on the placenta, first by keeping it on the ground and licking it, and then by grabbing it with her hands and chewing it (Fig. 1d). During placenta consumption, she touched her infant’s corpse and her own anogenital area. At 05:13 p.m., she stopped feeding on the placenta and started to sever the umbilical cord by biting it. A minute later, she detached the placenta from the umbilical cord and abandoned the corpse on the ground, while she was moving away holding the placenta with one hand. She then climbed to a tree at 15 m from the corpse location and continued consuming the placenta until 05:14 p.m., when she was out of sight again. At that stage, the female was at least 200 m away from the group but was vigilant because she often looked around and listened to vocalizations of her groupmates. At 05:30 p.m., we left the group at their sleeping site. The next morning, the female was seen for the last time and only for a few seconds. We assumed that she probably died the following day or soon after because she was never seen again.

During that postpartum phase, the female spent most of her time cleaning the infant, being vigilant and self-exploring (Table 1). The entire postpartum phase lasted at least 38 min, including 7 min and 18 s of video recording (Table 1).

### Infant’s description

The infant was a female and her corpse showed signs of abnormality, like a deformed head (Fig. 1e and Fig. 1f). Although we could not assess whether deformation was a consequence of the delivery process, the infant was clearly dead at the time of parturition. The infant’s CRL was approximately 19.3 cm.

## Discussion

Here, we describe the first daytime parturition of a stillborn in a natural population of mandrills. The described parturition lasted a minimum of 8 h and 25 min, from which we witnessed ca. 2 h and video recorded a total of 33 min and 44 s. Our report adds to three previous studies (9 stillbirths total) which reported stillbirth events in wild primates (Agoramoorthy et al. 1988; Nash 1974; Nguyen et al. 2017).

Stillbirths have been mainly studied in humans and provisioned primate populations (Bowden et al. 1967; Brandt and Mitchell 1973; Cho et al. 1985; Fretts 2005; Kaigaishi and Yamamoto 2023; Saiyed et al. 2018; Sesbuppha et al. 2008; Small 1982). Although some of the causes of these events have been identified (e.g., maternal or placental infection, congenital disorders, placental abnormalities, fetal and maternal trauma, asphyxia during birth and maternal life history traits such as advanced age, Fretts 2005; Saiyed et al. 2018; Sesbuppha et al. 2008; Schalabritz-Loutsevitch et al. 2008; Small 1982), most of the stillbirths have undetermined cause (e.g., 41% of stillbirths in humans, Fretts 2005; 61% of stillbirths in *Macaca fascicularis*, Sesbuppha et al. 2008).

Similarly, the cause of the reported stillbirth in this mandrill population is unknown. Some perinatal behaviors displayed by the parturient female matched with those observed in other wild and captive primate births, including postures during contractions (e.g., bipedal and tripedal squat position; Turner et al. 2010), the frequent investigation of the anogenital area (e.g., Solanki and Zothansiama 2013), the ingestion of the afterbirth (e.g., Peker et al. 2009) and the inability to follow groupmates’ movements (Duboscq et al. 2008). The delivery showed, however, a series of seemingly abnormal events: it occurred during daytime (in our study group 98.13% of births occurred at night, *N* = 214 birth dates known to the day, unpublished data), the partum phase was extremely long, the infant was born in vertex occiput anterior presentation (i.e., facing the backside of the mother), and probably as a consequence of this event, the parturient female died soon after delivery.

First, the partum phase lasted 65 min, which appears significantly longer than the “few seconds to few minutes” (Brandt and Mitchell 1973; Turner et al. 2010) duration described in other non-human primate females who successfully gave birth to healthy infants (e.g., Brandt and Mitchell 1973; Ding et al. 2013; Duboscq et al. 2008; Nakamichi et al. 1992; Peker et al. 2009; Yao et al. 2012). Indeed, mothers are extremely vulnerable during the partum phase, suggesting strong selection to shorten it (Ding et al. 2013). In addition, in free-ranging Japanese macaques (*Macaca fuscata*), births with short partum phases resulted in infants in better condition than births that lasted a long time (Turner et al. 2010). Generally, long partum phases are associated to infants delivered in an abnormal position (Brandt and Mitchell 1973; Bowden et al. 1967; Nash 1974; Trevathan 1988). Here, the infant was born in vertex occiput anterior presentation while non-human primates are generally born in vertex occiput posterior presentation (Trevathan 1988), a position that allows mothers to self-assist the delivery and the infant immediately after birth (Ding et al. 2013; Hirata et al. 2011; Trevathan 1988; Yao et al. 2012), increasing infant’s survival (Ding et al. 2013; Hirata et al. 2011). On the contrary, the vertex occiput anterior presentation forces the mother to pull the infant in the opposite natural flexion of its body, which can injure the infant, and does not promote the dilatation of the cervix efficiently (Trevathan 1988). In non-human primates, abnormal birth presentation often resulted in the death of the infant (Nguyen et al. 2017; Trevathan 1988). Yet, a multiparous wild mantled howling-monkey (*Alouatta palliata*) successfully delivered her infant, born in breech presentation, by self-assisting the delivery (Moreno et al. 1991). It is possible that the previous experience of the mantled howling-monkey female was crucial to ensure infant’s survival. The study female mandrill was primiparous and, if the infant was alive at the time of delivery, the occiput anterior presentation and maternal inexperience might have prolonged the duration of the partum phase, resulting in the infant’s death.

Second, the age of the female (ca. 5 years old) lies within the range of ages at first birth in the study females (3.16–6.73 years). Although we did not document the exact timing of pregnancy, its duration was estimated to be 5 months. The infant was thus probably born prematurely, as the mean pregnancy length in this population is substantially larger (163–190 days; Dezeure et al. 2022). Furthermore, the infant’s CRL was 19.3 cm, 4.5 cm less than the CRL at birth described in a semi-captive population of mandrills (Setchell et al. 2001). The infant’s head was also clearly deformed (Fig. 1e). In addition, the parturient female showed several episodes of vaginal bleeding one month before delivery. During the same birthing season, four other pregnant females bled during the last weeks of their pregnancy and, for two of them, their pregnancy swelling deflated shortly after, indicating pregnancy failure (unpublished data). These observations indicate that the infant was probably born dead although we cannot fully exclude the possibility that the delivery in itself was responsible for the infant’s death.

During the prepartum phase, we did not witness any interaction between the parturient female and other group members, although she was in the center of the group at that time. As parturition progressed, the female was unable to follow the group, and by the time of delivery, she was alone, about 200 m away from her groupmates. Finally, the parturient female was only observed for four seconds carrying her infant’s corpse, the most frequently reported primate maternal reaction to the infant’s death (Fernández-Fueyo et al. 2021), and a behavior also observed on some occasions in our study population (unpublished data). Except from that, she did not show any other behavior that could be described as maternal care. Whether this is a consequence of the long delivery process or of her inexperience is unknwon, emphasizing the need for further research into the complexities of such behaviors.

This report contributes to the limited literature on non-human primate births. A recent study further indicated the current lack of knowledge on human births as well, especially in non-industrialized countries (reviewed in Nguyen et al. 2017). Birthing events are yet crucial and challenge the survival of both mother and infant. Birth reports from wild populations are thus key to understand the evolutionary pressures influencing birth-related behaviors and the ecology of pregnancy and pregnancy failures.

### Supplementary Information

Below is the link to the electronic supplementary material.

Supplementary file1 (DOCX 32 kb)

## Data Availability

All data related to this article is included in this article.
