# Supplementary material for: Stillbirth of a mandrill (Mandrillus sphinx) in the wild: perinatal behaviors and delivery sequences
Source: Primates. 2023 Dec 22;65(2):75–80. doi: 10.1007/s10329-023-01112-6 (PMC10884356; doi:10.1007/s10329-023-01112-6)
Supplement: Supplementary file 1 — Supplementary file1 (DOCX 32 kb) [file 10329_2023_1112_MOESM1_ESM.docx]

| **TABLE S1:** Reported diurnal births and stillbirths in wild non-human primates. | | | | |
| --- | --- | --- | --- | --- |
| Species | Common name | N. of births | N. of stillbirths | References |
| *Alouatta belzebul* | Red-handed howler monkey | 2 | 0 | (Camargo and Ferrari 2007) |
| *Alouatta caraya* | Black and gold howler monkey | 4 | 0 | (Peker et al. 2009) |
| *Alouatta guariba* | Brown howler monkey | 1 | 0 | (Martins et al. 2015) |
| *Alouatta palliata* | Mantled howler monkey | 3 | 0 | (Dias 2005; Moreno et al. 1991; Nisbett and Glander 1996) |
| *Alouatta pigra* | Black howler monkey | 1 | 0 | (Cárdenas-Navarrete and Van Belle,2023) |
| *Alouatta seniculus* | Red howler monkey | 2 | 0 | (Sekulic 1982) |
| *Callicebus oenanthe* | Andean titi monkey | 1 | 0 | (DeLuycker 2014) |
| *Cercocebus galeritus* | Tana River Crested Mangabey | 1 | 0 | (Kinnaird 1990) |
| *Cercopithecus mitis stuhlmanni* | Blue monkey | 22 | 0 | (Brogan and Cords 2010; Cords and Gometz 2022) |
| *Erythrocebus patas* | Patas monkey | 7 | 0 | (Chism et al. 1983) |
| *Gorilla beringei* | Eastern gorilla | 3 | 0 | (Stewart 1977, 1984) |
| *Lemur catta* | Ring-tailed lemur | 10 | 0 | (Takahata et al. 2001) |
| *Macaca fuscata* | Japanese macaque | 2 | 0 | (Nakamichi et al. 1992; Nakamichi et al. 2004) |
| *Macaca nigra* | Crested black macaque | 1 | 0 | (Duboscq et al. 2008) |
| *Macaca sinica* | Toque macaque | 1 | 0 | (Ratnayeke and Dittus 1989) |
| *Nasalis larvatus* | Proboscis monkey | 1 | 0 | (Gorzitze 1996) |
| *Pan paniscus* | Bonobo | 1 | 0 | (Douglas 2014) |
| *Pan troglodytes* | Chimpanzee | 3 | 0 | (Fujisawa et al. 2016; Goodall and Athumani 1980; Kiwede 2000; Zamma et al. 2012) |
| *Papio anubis* | Olive baboon | 0 | 1 | (Nash 1974) |
| *Papio cynocephalus* | Yellow baboon | 1 | 0 | (Condit and Smith 1994) |
| *Papio hamadryas* | Hamadryas baboon | 1 | 0 | (Abegglen and Abegglen 1976) |
| *Pongo pygmaeus* | Bornean orangutan | 2 | 0 | (Galdikas 1982) |
| *Presbytis entellus* | Northern plains gray langur | 1 | 1 | (Agoramoorthy et al. 1988; Oppenheimer 1976) |
| *Rhinopithecus bieti* | Black-and-white snub-nosed monkey | 2 | 0 | (Ding et al. 2013; Li et al. 2020) |
| *Rhinopithecus roxellana* | Golden snub-nosed monkeys | 1 | 0 | (Yang et al. 2016) |
| *Saguinus imperator* | Emperor tamarin | 1 (twins) | 0 | (Windfelder 2000) |
| *Theropithecus gelada* | Gelada | 14 | 2 | (Dunbar and Dunbar 1974; Nguyen et al. 2017) |
| *Trachypithecus leucocephalus* | White-headed langur | 4 | 0 | (Pan et al. 2014; Yao et al. 2012) |
| *Trachypithecus pileatus* | Capped langur | 1 | 0 | (Kumar et al. 2005) |

| **TABLE S2:** Summary of behaviors exhibited by the parturient female. | |
| --- | --- |
| Behavior | Description |
| Carrying | Carrying the infant’s corpse |
| Cleaning | Investigating and cleaning the infant’s body by touching, licking and sniffing it, as well as licking and sniffing the female’s own fingers |
| Contraction | Undergoing a uterine contraction by tensing all muscles while in a bipedal squat position and raising the body. The female might also use the hands to hold on to branches and rise up |
| Exploration of umbilical cord | Investigation of the umbilical cord by touching, pulling, licking and sniffing it, as well as licking and sniffing the female’s own fingers |
| Foraging | Searching for food and/or feeding |
| Moving | Moving quadrupedally and changing location |
| Placentophagia | Consuming the afterbirth |
| Resting | Stationary standing, sitting or lying down, including movements to adjust the position |
| Self-exploring | Manual investigation of the anogenital area by touching, scratching and dabbing it, by licking and sniffing the female’s own fingers and by twisting the female’s own body to look at it. It also includes licking the birth fluids from the fur and the substrate, and investigating the head of the infant when it has emerged (but not been delivered) |
| Vigilance | Being alert to the movement of the group |

**Reproductive context**

Mandrills are seasonal breeders and most births occur during a six-month window, with an average birth peak in early January in the study population (data from March 2012 to Match 2020, see for details: Dezeure et al. 2022). The stillbirth event described in this report took place during the 2020-2021 birthing season where 68 births total occurred, of whom 66 infants survived their first week of life. The two infants that did not survive the neonatal period were a female born on February 15^th^ who died six days later for unknown reasons, and the stillborn female reported here. The 2020-2021 birth peak was on January 5^th^ (unpublished data), both births thus occurred, respectively 46 and 16 days after the birth peak. In the study population, females that conceive later in the breeding season are more likely to miscarry than females that conceive in the middle of the breeding season (Dezeure et al. 2022). Although we were not in the field at the time of conception that resulted in the reported stillbirth (due to the COVID-19 pandemic), the associated date of birth indicates that it probably occurred in the middle of the previous breeding season, suggesting no abnormal circumstances around this conception.

**References**

Abegglen H, Abegglen J-J (1976). Field observation of a birth in hamadryas baboons. Folia Primatol 26:54–56. <https://doi.org/10.1159/000155729>

Agoramoorthy G, Mohnot SM, Sommer V, Srivastava A (1988). Abortions in free ranging Hanuman langurs (*Presbytis entellus*) - A male induced strategy? Human Evolution 3:297–308. <https://doi.org/10.1007/BF02435859>

Brogan C, Cords M (2010). Daytime birth in a wild blue monkey. Afr Primates 7:61–63.

Camargo CC, Ferrari SF (2007). Observations of daytime births in two groups of red-handed howlers (*Alouatta belzebul*) on an Island in the Tucuruí reservoir in Eastern Brazilian Amazonia. Am J Primatol 69 (10):1075–1079. <https://doi.org/10.1002/ajp.20414>

Cárdenas-Navarrete A, Van Belle S (2023) Birth of a wild black howler monkey (*Alouatta pigra*) at an anthropogenic site. Primates 64:17–23. <https://doi.org/10.1007/s10329-022-01022-z>

Chism J, Olson DK, Rowell TE (1983). Diurnal births and perinatal behavior among wild patas monkeys: Evidence of an adaptive pattern. Int J Primatol 4(2):167-184. <https://doi.org/10.1007/BF02743756>

Condit VK, Smith EO (1994). Yellow baboon labor and parturition at the Tana River National Primate Reserve, Kenya. Am J Primatol 33(1):51–55.

Cords M, Gometz E (2022). The birth hour of mammals:Insights from intra-specific variation in wild blue monkeys. Curr Zool 68(5):499–506. https://doi.org/10.1093/cz/zoab088

DeLuycker AM (2014). Observations of a daytime birthing event in wild titi monkeys (*Callicebus oenanthe*): Implications of the male parental role. Primates 55(1):59–67. <https://doi.org/10.1007/s10329-013-0368-0>

Dezeure J, Charpentier MJE, Huchard E (2022). Fitness effects of seasonal birth timing in a long-lived social primate living in the equatorial forest. Anim Behav 185:113–126. <https://doi.org/10.1016/j.anbehav.2022.01.002>

Dias PAD (2005). Observation of parturition in the Mexican mantled howler monkeys (*Alouatta palliata*) on the Island of Agaltepec, Veracruz State, Mexico. Am J Primatol 65(1):93–98. <https://doi.org/10.1002/ajp.20100>

Ding W, Yang L, Xiao W (2013). Daytime birth and parturition assistant behavior in wild black-and-white snub-nosed monkeys (*Rhinopithecus bieti*) Yunnan, China. Behav Process 94:5–8. <https://doi.org/10.1016/j.beproc.2013.01.006>

Douglas PH (2014). Female sociality during the daytime birth of a wild bonobo at Luikotale, Democratic Republic of the Congo. Primates, 55(4), 533–542. <https://doi.org/10.1007/s10329-014-0436-0>

Duboscq J, Neumann C, Perwitasari-Farajallah D, Engelhardt A (2008). Daytime birth of a baby crested black macaque (*Macaca nigra*) in the wild. Behav Proces, 79(1), 81–84. <https://doi.org/10.1016/j.beproc.2008.04.010>

Dunbar RIM, Dunbar P (1974). Behaviour related to birth in wild gelada baboons (*Theropithecus gelada*). Behaviour, 50(1/2), 185–191.

Fujisawa M, Hockings KJ, Soumah AG, Matsuzawa T (2016). Placentophagy in wild chimpanzees (*Pan troglodytes verus*) at Bossou, Guinea. Primates, 57(2), 175–180. <https://doi.org/10.1007/s10329-016-0510-x>

Galdikas BMF (1982). Wild orangutan birth at Tanjung Puting reserve. Primates, 23(4), 500–510. <https://doi.org/10.1007/BF02373961>

Goodall J, Athumani J (1980). An observed birth in a free-living chimpanzee (*Pan troglodytes schweinfurthii)* in Gombe National Park, Tanzania. Primates, 21(4), 545–549. <https://doi.org/10.1007/BF02373843>

Gorzitze AB (1996). Birth-related behaviors in wild proboscis monkeys (*Nasalis larvatus*). Primates, 37(1), 75–78. https://doi.org/10.1007/BF02382922

Kinnaird MF (1990). Pregnancy, gestation and parturition in free-ranging tana river crested mangabeys (*Cercocebus galeritus galeritus*). Am J Primatol 22(4):285–289. <https://doi.org/10.1002/ajp.1350220408>

Kiwede ZT (2000). A live birth by a primiparous female chimpanzee at the Budongo Forest. Pan Africa News, 7(2):23–25.

Kumar A, Solanki GS, Sharma BK (2005). Observations on parturition and allomothering in wild capped langur (*Trachypithecus pileatus*). Primates 46(3):215–217. https://doi.org/10.1007/s10329-004-0121-9

Li Y-P, Zhong T, Huang Z-P, Pan R-L, Garber PA, Yu F-Q, Xiao W. (2020). Male and female birth attendance and assistance in a species of non-human primate (*Rhinopithecus bieti*). Behav Processes 181:104248. https://doi.org/10.1016/j.beproc.2020.104248

Martins V, Chaves ÓM, Neves MB, Bicca-Marques JC (2015). Parturition and potential infanticide in free-ranging *Alouatta guariba clamitans*. Primates 56(2):119–125. <https://doi.org/10.1007/s10329-015-0461-7>

Moreno LI, Salas IC, Glander KE (1991). Breech delivery and birth-related behaviors in wild mantled howling monkeys. Am J Primatol 23(3):197–199. <https://doi.org/10.1002/ajp.1350230306>

Nakamichi M, Imakawa S, Kojima Y, Natsume A (1992). Parturition in a free-ranging Japanese monkey (*Macaca fuscata*). Primates 33(3):413–418. <https://doi.org/10.1007/BF02381203>

Nakamichi M, Yamada K, Ohtsuka N, Imakawa S, Yasuda J, Shizawa Y (2004) Case reports on parturition and maternal behavior in a free-ranging group of Japanese monkeys at Katsuyama. Primate Res 20:31–43. https://doi.org/10.2354/psj.20.31

Nash LT (1974). Parturition in a feral baboon (*Papio anubis*). Primates 15(2):279–285. https://doi.org/10.1007/BF01742289

Nguyen N, Lee LM, Fashing PJ, Nurmi NO, Stewart KM, Turner TJ, Barry TS, Callingham KR, Goodale CB, Kellogg BS, Burke RJ, Bechtold EK., Claase MJ, Eriksen GA, Jones SCZ, Kerby JT, Kraus JB, Miller CM, Trew TH, … Venkataraman VV (2017). Comparative primate obstetrics: Observations of 15 diurnal births in wild gelada monkeys (*Theropithecus gelada*) and their implications for understanding human and nonhuman primate birth evolution. Am J Phys Anthropol 163(1):14–29. https://doi.org/10.1002/ajpa.23141

Nisbett R, Glander K (1996). Quantitative description of parturition in a wild mantled howling monkey: A case study of prenatal behaviors associated with a primiparous delivery. Brenesia 45:157–168.

Oppenheimer JR (1976). *Presbytis entellus*: Birth in a free-ranging primate troop. Primates 17(4):541–542. https://doi.org/10.1007/BF02382912

Pan W, Gu T, Pan Y, Feng C, Long Y, Zhao Y, Meng H, Liang Z, Yao M (2014). Birth intervention and non-maternal infant-handling during parturition in a nonhuman primate. Primates 55(4):483–488. https://doi.org/10.1007/s10329-014-0427-1

Peker S, Kowalewski MM, Pavé RE, Zunino GE (2009). Births in wild black and gold howler monkeys (*Alouatta caraya*) in Northern Argentina. Am J Primatol 71(3):261–265. <https://doi.org/10.1002/ajp.20643>

Ratnayeke AP, Dittus WPJ (1989). Observation of a birth among wild toque macaques (*Macaca sinica*). Int J Promatol 10(3):235–242. https://doi.org/10.1007/BF02735202

Sekulic R (1982). Birth in free-ranging howler monkeys *Alouatta seniculus*. Primates 23(4):580–582. <https://doi.org/10.1007/BF02373970>

Stewart KJ (1977). The birth of a wild mountain gorilla (*Gorilla gorilla beringei*). Primates 18(4):965–976. https://doi.org/10.1007/BF02382947

Stewart KJ (1984). Parturition in wild gorillas: Behaviour of mothers, neonates, and others. Folia Primatol 42(1):62–69. <https://doi.org/10.1159/000156144>

Takahata Y, Koyama N, Miyamoto N, Okamoto M (2001). Daytime deliveries observed for the ring-tailed lemurs of the berenty reserve, Madagascar. Primates 42(3):267–271. <https://doi.org/10.1007/BF02629642>

Windfelder TL (2000). Observations on the birth and subsequent care of twin offspring by a lone pair of wild emperor tamarins (*Saguinus imperator*). Am J Primatol 52(2):107–113. https://doi.org/10.1002/1098-2345(200010)52:2<107::AID-AJP5>3.0.CO;2-M

Yang B, Zhang P, Huang K, Garber PA, Li B-G (2016). Daytime birth and postbirth behavior of wild *Rhinopithecus roxellana* in the Qinling Mountains of China. Primates 57(2):155–160. https://doi.org/10.1007/s10329-015-0506-y

Yao M, Yin L, Zhang L, Liu L, Qin D, Pan W (2012). Parturitions in wild white-headed langurs (*Trachypithecus leucocephalus*) in the Nongguan Hills, China. Int J Primatol 33 (4):888–904. https://doi.org/10.1007/s10764-012-9625-0

Zamma KS, Sakamaki , Kitopeni R (2012). A wild chimpanzee birth at Mahale. Pan Africa News 19(1):3–5.
